# Nutrition Strategy and Life Style in Polycystic Ovary Syndrome—Narrative Review

**DOI:** 10.3390/nu13072452

**Published:** 2021-07-18

**Authors:** Małgorzata Szczuko, Justyna Kikut, Urszula Szczuko, Iwona Szydłowska, Jolanta Nawrocka-Rutkowska, Maciej Ziętek, Donatella Verbanac, Luciano Saso

**Affiliations:** 1 Department of Human Nutrition and Metabolomics, Pomeranian Medical University in Szczecin, Broniewskiego 24 St, 71-460 Szczecin, Poland; justyna.kikut@pum.edu.pl (J.K.); urszula.szczuko@gmail.com (U.S.); 2 Department of Gynecology, Endocrinology and Gynecological Oncology, Pomeranian Medical University in Szczecin, Unii Lubelskiej 1 St, 71-256 Szczecin, Poland; iwona.szydlowska@pum.edu.pl; 3 Department of Perinatology, Obstetrics and Gynecology Pomeranian Medical University in Szczecin, Siedlecka 2 St, 72-010 Police, Poland; jolanta.nawrocka@pum.edu.pl (J.N.-R.); maciej.zietek@pum.edu.pl (M.Z.); 4 Department of Medical Biochemistry and Hematology, Faculty of Pharmacy and Biochemistry, University of Zagreb, A. Kovačića 1, 10000 Zagreb, Croatia; donatella.verbanac@pharma.unizg.hr; 5 Department of Physiology and Pharmacology “Vittorio Erspamer”, Sapienza University, P. le Aldo Moro 5, 00185 Rome, Italy; luciano.saso@uniroma1.it

**Keywords:** PCOS, reproduction, lifestyle, diet, sleep, supplementation, herbs supporting

## Abstract

Here we present an extensive narrative review of the broadly understood modifications to the lifestyles of women with polycystic ovary syndrome (PCOS). The PubMed database was analyzed, combining PCOS entries with causes, diseases, diet supplementation, lifestyle, physical activity, and use of herbs. The metabolic pathways leading to disturbances in lipid, carbohydrate, and hormonal metabolism in targeted patients are described. The article refers to sleep disorders, changes in mental health parameters, and causes of oxidative stress and inflammation. These conditions consistently lead to the occurrence of severe diseases in patients suffering from diabetes, the fatty degeneration of internal organs, infertility, atherosclerosis, cardiovascular diseases, dysbiosis, and cancer. The modification of lifestyles, diet patterns and proper selection of nutrients, pharmacological and natural supplementation in the form of herbs, and physical activity have been proposed. The progress and consequences of PCOS are largely modifiable and depend on the patient’s approach, although we have to take into account also the genetic determinants.

## 1. Introduction

Polycystic ovary syndrome (PCOS) is the most common female endocrinopathy, affecting as many as 15% to 18% of women of reproductive age [1]. The definition of PCOS changed in 2003, when representatives of the European Society of Human Reproduction and American Society of Reproductive Medicine met in Rotterdam, The Netherlands. Currently, it is defined as a heterogeneous group with different phenotypes, which pose challenges in its treatment [2]. It seems, however, that some dependences and the tendency of the occurrence of the similar metabolic disorders are comparable [3].

Many studies have shown that higher hormone levels, gut microbiome composition, and plasma metabolomics are new parameters related to the PCOS phenotypes [4]. The clinical phenotypes can change over the life span with weight gain, and can coexist in the same patient. Individualized treatment remains the main approach, but grouping the phenotypes and following therapeutic recommendations may also prove to be clinically suitable. Precise recommendations should be implemented long before metabolic complications occur, which is particularly important for women with PCOS as they are predisposed to developing endometrial and ovarian cancer [5,6]. Therefore, the therapeutic approaches aimed at using anti-inflammatory remedies in supplementing and supporting anticancer therapy are crucial. They can help in inactivating the cascade of the deteriorating signaling pathways. Through these, better survival, faster recovery, and the improvement of the patients’ quality of life can be achieved.

### 1.1. Physiological Basis

**Figure 1:**
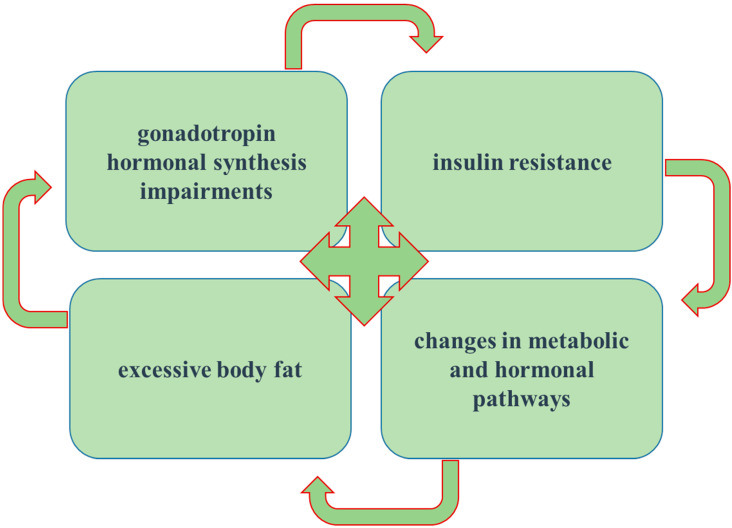
Main pathophysiological basis of polycystic ovary syndrome (PCOS)-disorders of gonadotropin hormonal synthesis, the appearance of insulin resistance, the influence of the present excessive body fat and oblique metabolic pathways involved in PCOS.

The four main causes of the physiological basis of PCOS include:

- disorders of gonadotropin hormonal synthesis;
- the appearance of insulin resistance;
- the influence of the present excessive body fat; and finally,
- the metabolic pathways involved in PCOS (the secretion and activity of insulin, encoding for steroidogenesis, and other metabolic and hormonal pathways) (Figure 1) [7].

Appropriate functioning of the mechanisms responsible for the maturation of the ovarian follicle and its ovulation depends on the proper physiological activity of three organs: the hypothalamus, pituitary gland, and ovaries.

The mechanisms of hormonal regulation in the hypothalamic-pituitary-ovarian system take place through the axes of negative feedback: long, short and ultra short feedback. In the suprachiasmatic nucleus of the hypothalamus there are neurons synthesizing gonadotropin-releasing hormone (GnRH), which is released into the pituitary portal circulation in the median eminence. GnRH release is regulated by a network of interconnected neurons. Gonadoliberin is an example of a hormone secreted in a pulsatile rhythm, and the frequency of this rhythm determines the type of gonadotropin released. A low frequency of gonadoliberin pulses results in the secretion of follicle-stimulating hormone (FSH), while a high frequency results in the secretion of luteinizing hormone (LH) from the anterior lobe of the pituitary gland. LH is responsible for the luteinization of the corpus luteum, i.e., the transformation of granulosa cells into theca lutein cells which produce progesterone. In turn, FSH stimulates ovarian follicle maturation and estrogen secretion in the granulosa cells of ovarian follicles. It also increases the activity of aromatase, the enzyme responsible for converting androgens (testosteron and androstendion) to estrogens. When the concentration of luteinizing hormone increases relative to FSH, excessive androgen production occurs, which is more common in women with PCOS [8].

Insulin, both directly and indirectly, affects the pathogenesis of PCOS. It acts synergistically with luteinizing hormone, increasing the production of androgens (theca cells) and decreasing the liver synthesis of the main binding testosterone protein (SHBG), which results in testosterone circulating in the unbound, active form [8]. Excess body fat is involved in the development of PCOS in many ways. Adipose tissue cells (adipocytes) produce peptide hormones like resistin and leptin, as well as some inflammatory cytokines (IL-beta, TNF-alpha) [9].

The activity of leptin affects the function of the hypothalamus–pituitary gland–ovary axis by modifying the secretion of GnRH, LH, and FSH. Leptin is a signal for the hypothalamus to release LH, causing the secretion of pituitary GnRH, as well. This can result in excessive androgen synthesis. Adipose tissue, by secreting pro-inflammatory factors such as mentioned cytokines, contributes to the development of inflammation in PCOS and an increased amount of free radicals caused by hyperglycemia; excess adipose tissue and androgens contribute to the formation of chronic inflammation in PCOS [8].

The various clinical symptoms of the disease indicate that many metabolic pathways participate in PCOS development, including: secretion and activity of insulin, with genes encoding for insulin receptor (IR), insulin (INS), and insulin-like growth factor (IGF) and its receptor; genes encoding for steroidogenesis; genes responsible for the activity of cytochrome P450 (CYP 17, CYP 11 alpha); and other metabolic and hormonal pathways, with genes for androgenic receptor (AR), LH receptor, leptin, and follistatin [10]. Moderate adherence to an anti-inflammatory dietary pattern and the low glycemic index (GI) and low-fat dietary pattern, have protective effect on the odds of developing PCOS [11,12].

### 1.2. Improvement in Metabolic Pathways

#### 1.2.1. Insulin Resistance

Weight gain mediates most of its direct medical sequelae through worsening insulin sensitivity.

Insulin resistance (IR) plays a key role in the development of metabolic dysfunction, including hypertension, dysglycemia, and dyslipidemia. A large amount of evidence supports a role of mitochondrial dysfunction in the development of IR, stimulated through ectopic fat deposition. Lipid-induced production of reactive oxygen species (ROS) within skeletal muscle promotes mitochondrial dysfunction and the development of IR [13]. Ultimately, IR underlies obesity-related conditions such as polycystic ovary syndrome (PCOS).

The cellular effects of insulin occur through two main post-receptor pathways: the phosphatidylinositol 3-kinase (PI3K) and the mitogen-activated protein kinase (MAPK) pathways [14]. The PI3K pathway regulates cellular intermediary metabolism, whereas the MAPK pathway controls growth processes and mitoses [14]. AKR1C3 expression in adipocytes leads to the occurrence of insulin resistance and hyperinsulinemia, then drives a vicious circle of intra-adipose androgen activation, lipid accumulation, and hyperinsulinemia [15]. Kauffman et al. suggested that ethnicity plays an additive effect on insulin resistance in PCOS. Mexican American women showed significantly higher insulin resistance compared with Caucasian American women [16].

#### 1.2.2. Oxidative Stress and Chronic Inflammation

The association between body weight and IR is mediated through inflammatory pathways [17]. Obesity causes changes in the release of key cytokines and adipokines, which in turn manifest in paracrine and endocrine effects. The increased levels of leptin and plasminogen activator inhibitor-1 and the reduced release of adiponectin result in a generalized low-grade inflammatory response. This process is mediated by macrophages and other immune cells.

Increases in ROS generation, p47phox gene expression, and circulating thiobarbituric acid-reactive substances (TBARS) occur in PCOS in response to saturated fat ingestion independent of obesity. A diet rich in simple sugars, as well as saturated fatty acids additionally enhances the production of ROS by different mechanisms, including the influence on gut microbiota [18]. Circulating mononuclear cells and excess adipose tissue are separate and distinct contributors to oxidative stress in this disorder [19]. Lipid-stimulated oxidative stress may be a key driver of insulin resistance and hyperandrogenism in PCOS. Excess adipose tissue is a contributor to the pro-oxidant burden and an additional regulator of insulin action [19]. Moreover, the chronic exposure to androgens results in an increase in oxidative stress in islet cells, inducing mitochondrial dysfunction [20,21].

Superoxide is a ROS produced when NADPH is oxidized by membrane-bound NADPH oxidase [22]. Dysregulated ROS production from NADPH oxidase has been implicated in a variety of cardiovascular disorders, including endothelial dysfunction, atherosclerosis, and hypertension, which are observed in women with PCOS [23]. Peroxide-induced oxidative stress activates nuclear factor-κB (NF-κB), which is a cardinal inflammatory signal that increases tumor necrosis factor (TNF)-α gene transcription [24]. Oxidative stress in response to saturated fat ingestion is an intermediate step in stimulating TNF-*α* secretion from circulating leukocytes [19,25]. In our investigations, we also showed that women with PCOS exhibit increased TNF-α synthesis [4]. Women with PCOS with normal and low levels of androgens measured by the level of testosterone and free androgen index (FAI) were more susceptible to the development of oxidative stress and inflammation induced by TNF-α [26].

#### 1.2.3. Anticancer Protection

Many studies have targeted the inactivation of the transcription factor (NRF2) as a therapeutic approach in various types of cancer [27]. NRF2 was first recognized in anticancer research as an inducer of several antioxidant enzymes. It can protect cells and tissues against many types of toxicant that interrupt essential biochemical processes and carcinogens by increasing the expression of cytoprotective genes [28]. NRF2 can act as a double-edged sword, being able to mediate both tumor-suppressive or pro-oncogenic functions depending on the specific biological context of its activation [29]. In line with this principle, the controlled activation of NRF2 might reduce the risk of cancer initiation and development in normal cells by scavenging ROS and by preventing genomic instability through decreased DNA damage. In contrast, already transformed cells with constitutive or prolonged activation of NRF2 signaling might represent a major clinical hurdle and exhibit an aggressive phenotype characterized by therapy resistance and unfavorable prognosis, requiring the use of NRF2 inhibitors [29].

It has been found that there are at least three pathways controlling the stability of NRF2. The first one depends on the cytosolic repressor KEAP1 [30]; the second is connected with the β-transducin repeat-containing protein (β-TrCP) [31]; while the third is related to the protein HRD1, which is an E3 ubiquitin ligase associated with the endoplasmic reticulum [32].

The abnormal activation of the NRF2/KEAP1 pathway promotes cancer development [33], metastasis formation [34], and even resistance to ovarian cancer therapy [35]. Mutations in the KEAP1 gene induce the hyper activation of the NRF2/KEAP1 pathway. Notably, KEAP1 missense or nonsense mutations were reported in endometrial carcinomas [36], as well as gall bladder [37], breast [38,39], cervical [40], and ovarian [41,42] cancers. MicroRNA miR-141 was the first-identified miRNA to directly repress KEAP1 levels in ovarian carcinoma cell lines [43].

### 1.3. Gut Microbiota Dysbiosis

The structural and functional dysbiosis of the gut microbiota in high-fat diet (HFD)-induced obesity was demonstrated in a mouse model [44]. The microbiota, through its metabolites, has multiple and complex effects on appetite, lipids, and carbohydrate metabolism and may influence body weight [44,45]. The gut microbiota can regulate about 10% of the host’s transcriptome and genes involved in the immune response, proliferation, and metabolism [46]. Interest in dietary fiber, gut fermentation, and probiotics has led to extensive research in this field [47]. The role of dietary fiber was demonstrated to modulate gut microbiota dysbiosis in patients with type 2 diabetes [48]. The growth of *Bifidobacteria* correlates with insulin secretion and increased glucose tolerance, regulates IR, and helps reduce inflammation. Short-chain fatty acids (SCFAs) such as acetate and butirate produced by the beneficial gut flora influence glycemia through glucagon-like peptide 1 (GLP-1) and pancreatic polypeptide (PPY), which are intestinal hormones [45]. The hormone PYY is a peptide that acts as a paracrine substance to stimulate the feelings of satiety or hunger in the control center [49]. Due to the absolute role of metabolites such as SCFAs in the metabolism of lipids and carbohydrates, ensuring the good condition of the microbiota is one of the therapeutic goals [50] in combating inflammation at local and systemic levels [51], as well as infections of the urogenital tract [52].

## 2. Lifestyle Changes

Lifestyle change is the first line of treatment for the management of women with PCOS but is not an alternative to its pharmacological treatment [7]. Regular physical activity, maintaining appropriate body weight, following healthy dietary patterns and avoiding tobacco use is vital in prevention and treatment of metabolic disorders, and is included in clinical guidelines for various conditions. Focusing on overall wellbeing and mental health is a personal choice, and while it is not an immediate fix, it is an important step towards a more fulfilling life.

Nutritional counseling for PCOS patients has been one of the treatment methods for many years. However, strict caloric restrictions do not produce the expected long-term effects [53,54], and the isocaloric diet did not significantly improve the biochemical and anthropometric parameters even in combination with physical activity [55].

### 2.1. Diet

Analysis of the impact of lifestyle modification related to the share of energy from macronutrients (protein, fat, and carbohydrates) showed no significant differences in the levels of the analyzed parameters. However, a significant factor in these changes was the reduction in the caloric content of the diet [56] and the introduction of a reduced-calorie diet with a low GI [57]. Low GI (LGI) diets decreased homeostatic model assessment for insulin resistance (HOMA-IR), fasting insulin, total and low-density lipoprotein (LDL) cholesterol, triglycerides, waist circumference, and total testosterone compared with high GI (HGI) diets without affecting fasting glucose, HDL cholesterol, weight, or the free androgen index [58]. In addition, the inclusion of the LGI diet, punitive restrictions, and/or physical activity, and the supplementation of omega-3 increased HDL, sex hormone binding globulin (SHBG) synthesis, and reduction in body fat [8]. Gonzales et al. found that saturated fat acid (SFA) ingestion stimulates increases in circulating TNF-*α* and peripheral leukocytic suppressor of cytokine-3 (SOCS-3) expression [25]. Therefore, eliminating SFA from the diets of these patients is imperative. Dietary α-linolenic acid-rich flaxseed oil exerted beneficial effects on polycystic ovary syndrome through the sex steroid hormones–microbiota–inflammation axis in rats, but other sources of α-linolenic acid will probably produce an equally good effect [59].

The effects of soluble dietary fiber on SCFAs were demonstrated. Fermentable fiber has positive metabolic benefits on the gut microbiome with subsequent release of SCFAs [60]. Diets with a low GI may influence appetite-regulating hormones including ghrelin and glucagon [12,61]. Low-GI meals reduced ghrelin and increased glucagon in women with PCOS [61]. High fructose consumption (HFC) synergistically aggravated endocrine but not metabolic changes in PCOS, suggesting that (HFC) might deteriorate endocrine-related phenotypes in PCOS [62]. A meta-analysis and systematic review showed that the LGI diet is an effective, acceptable, and safe intervention for relieving IR, and professional dietary advice should be offered to all PCOS patients [63,64].

It seems that another reduced-GI diet modification is the ketogenic diet, which limits the consumption of total carbohydrates in favor of plant-based fat. The ketogenic diet (KD) improves the menstrual cycle, reducing blood glucose and body weight, improving liver function, and treating fatty liver in women with PCOS and liver dysfunction who were obese [65]. Even more interesting results were reported by Paoli et al. after using the KD for 12 weeks in women with PCOS [66]. The anthropometric and body composition measurements revealed a significant reduction in body weight (−9.43 kg), body mass index (BMI; −3.35), and fat-free body mass (8.29 kg). A significant decrease in glucose and insulin blood levels was observed, together with a significant improvement in HOMA-IR scores. A significant decrease of triglycerides, total cholesterol and LDL were observed along with a rise in HDL levels. The LH/FSH ratio, LH total and free testosterone, and DHEAS blood levels were also significantly reduced. Estradiol, progesterone and SHBG increased. The Ferriman Gallwey Score was slightly, although not significantly, reduced [66]. There was no significant association between parameters of hirsutism and the visceral adiposity index (VAI). Hirsutism is unlikely to be due to visceral adipocyte dysfunction [67]. Therefore, in PCOS patients with advanced obesity and/or obesity accompanied by full-blown metabolic syndrome, the introduction of a ketogenic diet may provide even better results than a diet with a LGI. Nonetheless, a general conclusion is that by following the main principles of a healthy diet, the physiological homeostasis can be managed, as well as faster recovery from disease achieved.

### 2.2. Physical Activity

Exercise training in the management of PCOS is becoming more recognized and accepted among professionals in the health sector and the patients. Physical training potentiates the effects caused by insulin sensitivity through the optimization of glucose transport and metabolism [68].

A recent meta-analysis found that improvements in health outcomes are more dependent on exercise intensity than dose. The results from this analysis support the use of exercise and that vigorous intensity exercise may have the greatest impact on cardiorespiratory fitness, insulin resistance, and body composition [69]. Insulin resistance, measured using the HOMA-IR and BMI showed a significant decrease with moderate and high certainty (MD-0.57; 95% confidence interval (CI), −0.98 to −0.16, and *p* = 0.01; MD-1.90, 95% CI −3.37, −0.42, and *p* = 0.01), respectively [70]. Other authors in a systematic review found that vigorous aerobic exercise and resistance training to improve insulin sensitivity and androgen measurements are warranted for women with PCOS. [71]. The minimum aerobic activity per week should be 120 min [69].

### 2.3. Sleep

Mental health disorders are highly prevalent in PCOS cases, which are associated with significantly more frequently experienced states of anxiety and depression, as well as sleep disorders [72]. Sleep disorders impact the etiology and development of the anxiety and depression seen in PCOS, so treating sleep-related conditions should be an integral part of treating women with PCOS [72]. Sleep deprivation has been connected with increased risk of IR, obesity, and type 2 diabetes (T2D) [73,74,75]. Although incompletely understood, the factors that mediate IR in response to sleep deprivation, likely implicated centrally regulated autonomic pathways, endocrine responses (e.g., changes in the key appetite hormones ghrelin and leptin), and inflammatory status. Mice experiencing sleep fragmentation (SF) showed white adipose tissue (WAT) inflammation and worsened IR, which resulted from enhanced disruption to the colonic epithelial barrier [76] and “gut leakage” syndrome which leads to LPS mediated inflammation [51]. Thus, SF-induced metabolic alterations may be mediated in part by concurrent changes in the gut microbiota, thereby providing an opportunity for gut-microbiome-targeted therapeutics [76]. The main pineal gland hormone melatonin is involved in the regulation of the circadian rhythm. In recent years, it was observed that a reduction in the melatonin levels of follicular fluid occurs in PCOS patients [77]. Melatonin receptors in the ovary and intrafollicular fluid adjust sex steroid secretion at different phases of ovarian follicular maturation. Melatonin is a strong antioxidant and an effective free-radical scavenger, which protects ovarian follicles during follicular maturation [77].

Based on current knowledge, it is plausible to conclude that sleep disorders can be considered as one of the first symptoms leading to the weakening of the body’s protective properties and intensification of the pathways associated with insulin resistance in the course of PCOS.

### 2.4. Supplementation

The research showed that the vast majority of women with PCOS consume an improperly balanced diet, involving deficiencies in fiber, omega 3, calcium, magnesium, zinc, and vitamins (folic acid, vitamin C, vitamin B12, and vitamin D) [8]. An excess of nutrients was also noted in sucrase, sodium, total fats, saturated fatty acids, and cholesterol [8]. It was examined whether the deficiencies can be balanced with a correct calories-reduction diet with a lowered GI and it resulted positive regarding influence on the water-soluble vitamins [78,79]. In the case of most vitamin B, the increase in its supply with the diet led to the expected result in the form of its increased level in the plasma of women with PCOS. This effect was not observed for vitamin B3, and the levels of B2 and thiamine were not as satisfactory as in the case of the other, related vitamins [79]. It was documented that the insufficient supply of vitamin B3 is associated with the development of inflammatory conditions, leading to the associated diseases [80] as well as the increased risk of cardiovascular syndromes [81]. Women with PCOS may be treated with metformin, which normalizes glycemia, but its chronic intake is additionally associated with deficiencies in thiamine and cobalamin [82]. Therefore, it is a good idea to supplement with thiamine, which, by activating transketolase, contributing to the inhibition of mechanisms damaging blood vessels, reducing the risk of cardiovascular diseases [83,84].

While drawing attention to the potential properties of blood vessel protection in PCOS, supplementation with coenzyme Q10 also requires consideration. CoQ10 supplementation for 8 weeks had a beneficial effect on inflammatory and endothelial dysfunction markers in overweight and obese patients with PCOS [85].

When analyzing the available literature on supplementation in PCOS, attention should be paid to vitamin D, which increases insulin synthesis and release, increases insulin receptor expression, and increases insulin response to glucose transport [86]. Vitamin D indirectly influences carbohydrate metabolism by normalizing extracellular calcium and parathyroid hormone concentration. It also affects the expression of the genes of the metabolic pathways affecting systemic inflammation by inhibiting the synthesis of pro-inflammatory cytokines, which may contribute to the occurrence of IR [87]. Women with PCOS receiving 20,000 IU of cholecalciferol weekly benefited from improved carbohydrate metabolism. Decreases in fasting glucose, triglycerides, and estradiol were observed. Although no changes in androgen levels were observed, improvements in menstrual frequency were noted [88]. Combined magnesium, zinc, calcium, and vitamin D supplementation in another study led to a significant reduction in hirsutism and total testosterone compared with the placebo, but supplementation did not affect SHBG levels or the free androgen index (FAI) [89]. Conversely, the combination of vitamin D and fish oil reduced the parameters of inflammation in the body (serum C-reactive protein (CRP), downregulation of interleukin (IL)-1 genes) and total testosterone levels and has beneficial effect on mental health parameters measured by Beck’s Depression Questionnaire [90].

Current results showed that myo-inositol is as effective as metformin in improving the clinical and metabolic profile of women with PCOS and the metabolic disorders associated with diabetes [91]. However, the administration of metformin is associated with side effects that are not experienced with inositol [92]. Inositol increases insulin sensitivity, normalizes androgens in the blood, improves glycemia, and affects numerous features of metabolic syndrome [93,94]. PCOS appears to involve increased epimerization of myo-inositol (MI) to d-chiro-inositol (DCI) in the ovary by insulin, the consequence of which is overproduction of DCI and deficiency of MI, which in turn affects the disturbance of FSH signaling and deterioration of the quality of oocytes [95]. Inositols (both isomers, both given separately and in combination) also have the potential to restore spontaneous ovulation and improve fertility in women with PCOS. An analysis of the literature showed supplementation with inositol as being a safe and, importantly, effective form of PCOS therapy, improving the development of ovarian follicles, oocyte maturation, and stimulation of pregnancy [96].

As in traditional medicine, natural substances such as isoquinoline alkaloids have been used to regulate the synthesis of androgens and the metabolism of lipids and carbohydrates, the introduction of berberine in patients with PCOS has been considered [97,98,99]. As with metformin, the beneficial metabolic effects of berberine in type II diabetes are related to the activation of adenosine monophosphate-activated protein kinase (AMPK). Berberine has good hypoglycemic and hypolipidemic effects, reduces body weight, and is an effective insulin sensitizer [100]. It also reduces the synthesis of steroid hormones and the expression of ovarian aromatase by acting on the hypothalamic–pituitary–ovarian axis, and improves the ovulation rate and the regulation of menstruation, thus increasing the pregnancy and live birth rates. In addition, studies showed that even with long-term use of berberine, its side effects are transient and mild (constipation, nausea) [101], which suggests that berberine may be a safe and promising compound for the treatment of PCOS patients [98,102].

Chromium is the basic element involved in the metabolism of carbohydrates and lipids; therefore, it has become one of the most commonly consumed dietary supplements in the USA [103]. The indications for its supplementation were once very broad; however, chrome is currently one of the most controversial components by which its influence is strongly undermined [104,105]. It was argued that it is not an essential micronutrient, but has potential benefits and/or side effects. By enhancing the insulin signaling pathway, increasing the activity of AMPK, and increasing cellular glucose uptake, it has a beneficial effect in PCOS patients in improving diabetes [106]. Decreases in the expressions of 3β-hydroxysteroid dehydrogenase and 17β-hydroxysteroid dehydrogenase were identified in adipose tissue, which were related to dehydroepiandrosterone [107].

The research, and the available literature, show that supplementation with zinc and selenium to counter deficiencies may be indicated in the case of at least some patients with PCOS. Due to intracellular signaling and structural functions, zinc plays a role in lipid and glucose metabolism and fertility [108]. Low zinc intake in obese people is associated with hyperinsulinemia, increased low-grade inflammation, and a worsened lipid profile. In addition, zinc ions can act in an insulin-mimetic manner in adipocytes, stimulating lipogenesis and glucose transport through the translocation of glucose transporter 4 (GLUT4) to the plasma membrane [109]. Zinc deficiency may play a significant role in the pathogenesis of PCOS and may be a prognostic marker of PCOS. Studies showed that the average serum zinc levels of PCOS patients are significantly lower compared with healthy controls [110]. In addition, serum zinc levels were shown to be lower in PCOS patients with impaired glucose tolerance than in PCOS patients with normal glucose tolerance. [110]. Selenium is associated with a lower level of CRP. It has anti-inflammatory and antioxidant properties [111]. Finally, it is necessary to supplement the omega-3 fatty acids, which tend to lack in the diet of PCOS women. However, with the balanced diet, supplementation can be regarded as a seasonal intervention [112]. Polyunsaturated fatty acids (PUFAs) enhance the reproductive performance in PCOS by increasing the expression of steroidogenesis enzymes, which are related to hormone secretion and ovarian functions, and the protein levels of CYP51, CYP19, StAR, and 3β-HSD [113]. In summary, supplementing the diet is an individual subject that requires dietary consultation with the patient, and its active participation and compliance is desirable for the overall improvement of the metabolic equilibrium. A properly balanced diet and a healthy lifestyle should be the first element of PCOS therapy.

### 2.5. Herbs Supporting Treatment

A balanced diet to support insulin management is the most important treatment for PCOS; drinking infusions of some herbs would therefore be a very good complement to the therapy, such as *Aloe vera*, cinnamon (*Cinnamomum verum*), green tea (*Camellia sinensi*), and chamomile (*Matricaria chamomilla*), and white mulberry (*Morus alba*) [114]. There are medical herbs can affect the lipid profile, blood glucose, and IR [115]. Because these herbs have properties of regulating lipid and carbohydrate metabolism they can be used by all phenotypes of PCOS women. Several of the herbs also have endocrine properties, these were the ones mentioned earlier: green tea [116] and marjoram (*Maiorana hortensis*) are some of the herbs whose effects include improvements in hormonal levels, ovaries weight, insulin sensitivity, antioxidants, and anti-inflammatory parameters [117,118].

Another group of herbs is indicated especially for women with PCOS with biochemical evidence of increased levels of androgens: green mint (*Mentha spicata* L.), which has an antiandrogenic effect and restores follicular development in ovarian tissue [119,120]; licorice smooth (*Glycyrrhiza glabra*) has been used in the treatment of PCOS because of its antiandrogen and estrogen-like activity. Licorice root appears to be effective in reducing excess testosterone as it blocks the conversion of androstenedione. Glycyrrhetinic acid and metabolites block 11 beta-hydroxysteroid dehydrogenase type 2 and bind mineralocorticoid receptors directly, acting as agonists [121,122]. However, licorice is not a flawless solution, having the potential to induce hypertension, hypokalemia, and metabolic alkalosis [123]. People with high cortisol levels should, therefore, avoid this preparation. The available literature suggests a role of herbal drugs in the action against 5-alpha-reductase enzyme, inhibiting it and reducing hair loss [124]. *Serenoa repens, Camellia sinensis, Rosmarinus officinalis*, and *Glycyrrhiza glabra* can also lower androgen levels and inhibit androgenetic alopecia [124]. *Vitex agnus-castus* is a good regulator of the menstrual cycle and has been used in traditional medicine for centuries [125]. The best-studied dietary phytoestrogens are the flaxseed lignans [126]. The lignan content of flax-seed (*Linum usitatissimum*) may alter the activity of key enzymes involved in estrogen synthesis (e.g., aromatase) to modulate relative levels of circulating sex hormones and their metabolites [127].

Turmeric (*Curcuma longa*), and specifically curcumin, is a biologically active phytochemical ingredient [128,129]. Curcumin seems to be an efficient reducer of oxidative-stress-related complications in patients with PCOS [130,131]. Moreover, curcumin attenuates proangiogenic and proinflammatory factors in human eutopic endometrial stromal cells through the NF-κB signaling pathway [132]. Nettle (*Urtica dioica)* is a multipurpose herb in medicine for which some antioxidative, anti-inflammatory, antimutation, and antitumor properties were identified [133,134]. The flavonoids are a family of compounds with antioxidant activities that can modify specific enzymes, so they can inactivate some agents such as nitrite peroxide and hydroxide radicals [135].

Ultimately, in advanced PCOS with accompanying disease associated with metabolic syndrome and the steatosis of internal organs (especially non-alcoholic fatty liver disease), herbs and their extracts with proven properties should be considered for their hepatoprotective activities [136]. These substances include the silymarin contained in milk thistle (*Silybum marianum*) [137,138] and sesquiterpenes and antioxidant-active ingredients in artichoke (*Cynara Cardunculus*) extract [139,140]. Dandelion (*Taraxacum officinale*) and its component taraxasterol may silence the gene of SIRT1, preventing the disruption of hepatic cells [141]. Black cumin (*Nigella sativa*) also has similar properties, which should be included in the diet of obese PCOS patients [142]. To summarize, herbs and the substances they contain offer many possibilities for interventions supporting the treatment of PCOS at various stages of disease. The selection of the appropriate mixture may be individualized depending on the occurrence of symptoms. Summary information has been added in Table 1.

## 3. Conclusions

The analysis of metabolic symptoms occurring in the course of PCOS points to the need for a multidirectional therapeutic approach. The metabolic pathways leading to the abnormalities are presented, which requires focusing on the improvement of parameters related to fertility, hirsutism, the occurrence of carbohydrate-lipid disturbances and the reduction of insulin resistance. One of the most important pathways for blocking carcinogenesis is presented. It has been shown that significant improvement of these parameters depends on modifiable factors related to the improvement of lifestyle, the introduction of a diet, especially a low-calorie diet with reduced GI, normalization of sleep and the introduction of daily physical activity. In addition, supplementing the diet with antioxidants and herbs seems to be highly effective in combating the chronic inflammation (*Curcuma longa*), improving liver steatosis (*Silybum marianum, Nigella sativa*) and the frequently occurring intestinal dysbiosis (probiotic therapy). Conducting our own research in this area, we examined how increasing the supply of vitamins and minerals with the diet affects the supply of these components in patients, so we also searched the literature and described suggested supplementation (inositol, thiamine, coenzyme Q10, vitamin D, zinc, selenium). Undoubtedly there is a need for further research to be undertaken to determine the efficacy and applicability of the ingredients described as a support for traditional PCOS management.

## 4. Methods of Searching

In this study, we reviewed the literature focused on PCOS therapy, unrelated to medical therapy, by searching the records of international PubMed and Embase (Elsevier) databases from the last 20 years.

All articles collected through the electronic search process used in this article were reviewed from the abstract. Articles unrelated to the main topic, duplicate papers in both databases (PubMed and Embase), and conference abstracts were excluded from the review process. Only articles published in English were considered.

The main core of the issue was the authors’ own 10 years of experience and research in this patient group. From the authors’ own studies, those that corresponded sequentially to the intervention steps discussed were selected. The physiological basis was discussed (searching the database for PCOS and insulin resistance or chronic inflammation or endocrine disorders or cancer or microbiota). Lifestyle changes were then discussed. Studies that examined the association between PCOS and diet or supplementation (pcos + inositol; PCOS + berberine; PCOS + vitamin D; PCOS + chromium; PCOS + zinc; PCOS + selenium; PCOS + melatonin) or adjunctive herbs were included in the review. In the case of duplication of information in publications, those that contribute most to the main topic were selected.

## Figures and Tables

**Table 1:** Table summarizing described interventions of herbs and their effects.

A Symptom Accompanying PCOS	Diet	Physical Activity	Sleep Regulation	Supplementation	Microbiota	Herbs
Hirsutism	reduced diet [26,44,45,54,58]			magnesium, zinc, calcium [89,108,109,110,111], and vitamin D [86,87,88,89,90], myo-inositol [93,94,95,96]		green mint [120,121], licorice smooth [122], *Serenoa repens*, *Camellia sinensis, Rosmarinus officinalis,* and *Glycyrrhiza glabra*
The androgens levels	diet with reduced GI and calorie [26,44,45,54,58], Ketogenic diet [64]			magnesium, zinc, calcium [89,108,109,110,111], and vitamin D [86,87,88,89,90], berberine [97,98,99,100,101,102], chromium [105,106,107], zinc [110]		green mint [120,121], licorice smooth [122], *Serenoa repens, Camellia sinensis, Rosmarinus officinalis,* and *Glycyrrhiza glabra* [124]
Ovulation disorders	diet with reduced GI and calorie [26,44,45,54,58], Ketogenic diet [64]			vitamin D [86,87,88,89,90], myo-inositol [97,98] berberine [99], zinc [108], PUFAs [112,113]		green mint [120,121], licorice smooth [121], *Vitex agnus-castus* [124], flax-seed [59,125,126]
Fat mass reduction	high-fiber diet with reduced GI and calorie [28,46,47,56,60], ketogenic diet [64] elimination SFA [22,58]	daily physical activity [68,69,70,71]	improving sleep [72,73,74,75,76,77]		microbiota and metabolites [46,47]	
Carbohydrate metabolism disorders	high-fiber diet with reduced GI and calorie [26,44,45,54,58], ketogenic diet [64]	daily physical activity [68,69,70,71]	improving sleep [72,73,74,75,76,77]	vitamin B1 [82,83,84], vitamin D [86,87,88,89,90], myo-inositol [91,92,93,94,95,96], berberine [97,98,99,100,101,102], chromium [105,106,107], zinc [109]	SCFA [47,52], microbiota and metabolites [50]	Aloe vera, cinnamon green tea [115], chamomile and white mulberry [117]
Insulin resistance	high-fiber diet with reduced GI and calorie [26,44,45,54,58], elimination SFA [22,58]	daily physical activity [71,72,73,74]	improving sleep [72,73,74,75,76,77] melatonin [77]	vitamin D [86,87,88,89,90], myo-inositol [91,92,93,94,95,96], berberine [97,98,99,100,101,102]	Bifidobacteria [45,50]	Aloe vera, cinnamon, green tea, chamomile and white mulberry [117]
Lipids metabolism disorders	high-fiber diet with reduced GI and calorie [26,44,45,54,58], elimination SFA [25,60]	daily physical activity [68,69,70,71]		omega 3 [112,113], berberine [97,98,99,100,101,102], zinc [110]	SCFA [47,52]; microbiota and metabolites [50]	milk thistle [137,138] artichoke extract [139,140]. Dandelion [141], Black cumin [142]
Steatosis of organs-liver profile	high-fiber diet with reduced GI and calorie [46,47,56,60]			silymarin [137,138], sesquiterpenes [139,140], taraxasterol [141]		milk thistle [137,138] artichoke extract [139,140]. Dandelion [141], Black cumin [142]
Cardiovascular diseases	high-fiber diet with reduced GI and calorie [46,47,56,60]	intensity exercise [72]		α-linolenic acid [59], vitamin B3 [80,81], vitamin B1 [82,83,84], coenzyme Q10 [85]		
Intestinal dysbiosis	high-fiber diet [49,50]				Bifidobacteria [45,50]	
Chronic inflammation	high-fiber diet with reduced GI and calorie [28,46,47,56,60]		melatonin [79]	α-linolenic acid [59], vitamin B3 [80,81], coenzyme Q10 [85], vitamin D [88,89], selenium [112], flavonoids [135]	Bifidobacteria [45,50]	Green tea and Marjoram [117,118,119], Turmeric [128,129,130,131], Nettle [133,134], milk thistle [137,138] Artichoke extract [139,140]. Dandelion [141], Black cumin [142]
Limiting predisposition to cancer	elimination SFA [25,27]; high-fiber diet [49,50]			α-linolenic acid [59]		Turmeric [128,129,130,131], Nettle [133,134]
Mental health disorders		daily physical activity [71,72,73,74]	improving sleep [75]	vitamin D [86,87,88,89,90], omega 3 (fish oil) [72,90]		

SCFA—short-chain fatty acids; GI—glycemic index; SFA—saturated fat acids; PUFA—Polyunsaturated fatty acid.

## Data Availability

The study did not report any data.

## References

[1] Fauser B.C.J.M., Tarlatzis B.C., Rebar R.W., Legro R.S., Balen A.H., Lobo R., Carmina E., Chang J., Yildiz B.O., Laven J.S.E. (2012). Consensus on Women’s Health Aspects of Polycystic Ovary Syndrome (PCOS): The Amsterdam ESHRE/ASRM-Sponsored 3rd PCOS Consensus Workshop Group. Fertil. Steril..

[2] Zuo M., Liao G., Zhang W., Xu D., Lu J., Tang M., Yan Y., Hong C., Wang Y. (2021). Effects of Exogenous Adiponectin Supplementation in Early Pregnant PCOS Mice on the Metabolic Syndrome of Adult Female Offspring. J. Ovarian Res..

[3] Szczuko M., Zapałowska-Chwyć M., Maciejewska D., Drozd A., Starczewski A., Stachowska E. (2017). Significant Improvement Selected Mediators of Inflammation in Phenotypes of Women with PCOS after Reduction and Low GI Diet. Mediat. Inflamm..

[4] Ma L., Cao Y., Ma Y., Zhai J. (2021). Association between hyperandrogenism and adverse pregnancy outcomes in patients with different polycystic ovary syndrome phenotypes undergoing in vitro fertilization/intracytoplasmic sperm injection: A systematic review and meta-analysis. Gynecol. Endocrinol..

[5] Martini A.E., Healy M.W. (2021). Polycystic Ovarian Syndrome: Impact on Adult and Fetal Health. Clin. Obs. Gynecol..

[6] Hong G., Wu H., Ma S.-T., Su Z. (2020). Catechins from Oolong Tea Improve Uterine Defects by Inhibiting STAT3 Signaling in Polycystic Ovary Syndrome Mice. Chin. Med..

[7] Del Pup L., Cagnacci A. (2021). IMPROVE Lifestyle in Polycystic Ovary Syndrome: A Systematic Strategy. Gynecol. Endocrinol..

[8] Szczuko M., Skowronek M., Zapałowska-Chwyć M., Starczewski A. (2016). Quantitative Assessment of Nutrition in Patients with Polycystic Ovary Syndrome (PCOS). Rocz. Panstw. Zakl. Hig..

[9] Makki K., Froguel P., Wolowczuk I. (2013). Adipose Tissue in Obesity-Related Inflammation and Insulin Resistance: Cells, Cytokines, and Chemokines. ISRN Inflamm..

[10] Dniak-Nikolajew A. (2012). Zespół Policystycznych Jajników Jako Przyczyna Niepłodności Kobiecej [Polycystic Ovary Syndrome as a Cause of Female Infertility]. Położna Nauka I Prakt..

[11] Panjeshahin A., Salehi-Abargouei A., Anari A.G., Mohammadi M., Hosseinzadeh M. (2020). Association between Empirically Derived Dietary Patterns and Polycystic Ovary Syndrome: A Case-Control Study. Nutrition.

[12] Szczuko M., Zapalowska-Chwyć M., Drozd R. (2019). A Low Glycemic Index Decreases Inflammation by Increasing the Concentration of Uric Acid and the Activity of Glutathione Peroxidase (GPx3) in Patients with Polycystic Ovary Syndrome (PCOS). Molecules.

[13] Di Meo S., Iossa S., Venditti P. (2017). Skeletal Muscle Insulin Resistance: Role of Mitochondria and Other ROS Sources. J. Endocrinol..

[14] Barber T.M., Kyrou I., Randeva H.S., Weickert M.O. (2021). Mechanisms of Insulin Resistance at the Crossroad of Obesity with Associated Metabolic Abnormalities and Cognitive Dysfunction. Int. J. Mol. Sci..

[15] Kempegowda P., Melson E., Manolopoulos K.N., Arlt W., O’Reilly M.W. (2020). Implicating Androgen Excess in Propagating Metabolic Disease in Polycystic Ovary Syndrome. Adv. Endocrinol. Metab..

[16] Kauffman R.P., Baker V.M., Dimarino P., Gimpel T., Castracane V.D. (2002). Polycystic Ovarian Syndrome and Insulin Resistance in White and Mexican American Women: A Comparison of Two Distinct Populations. Am. J. Obs. Gynecol..

[17] Shoelson S.E., Herrero L., Naaz A. (2007). Obesity, Inflammation, and Insulin Resistance. Gastroenterology.

[18] Fajstova A., Galanova N., Coufal S., Malkova J., Kostovcik M., Cermakova M., Pelantova H., Kuzma M., Sediva B., Hudcovic T. (2020). Diet Rich in Simple Sugars Promotes Pro-Inflammatory Response via Gut Microbiota Alteration and TLR4 Signaling. Cells.

[19] González F., Considine R.V., Abdelhadi O.A., Acton A.J. (2019). Oxidative Stress in Response to Saturated Fat Ingestion Is Linked to Insulin Resistance and Hyperandrogenism in Polycystic Ovary Syndrome. J. Clin. Endocrinol. Metab..

[20] Wang H., Wang X., Zhu Y., Chen F., Sun Y., Han X. (2015). Increased Androgen Levels in Rats Impair Glucose-Stimulated Insulin Secretion through Disruption of Pancreatic Beta Cell Mitochondrial Function. J. Steroid Biochem. Mol. Biol..

[21] Liu S., Navarro G., Mauvais-Jarvis F. (2010). Androgen Excess Produces Systemic Oxidative Stress and Predisposes to Beta-Cell Failure in Female Mice. PLoS ONE.

[22] Jiang F., Zhang Y., Dusting G.J. (2011). NADPH Oxidase-Mediated Redox Signaling: Roles in Cellular Stress Response, Stress Tolerance, and Tissue Repair. Pharm. Rev..

[23] Bedard K., Krause K.-H. (2007). The NOX Family of ROS-Generating NADPH Oxidases: Physiology and Pathophysiology. Physiol. Rev..

[24] Evans J.L., Goldfine I.D., Maddux B.A., Grodsky G.M. (2002). Oxidative Stress and Stress-Activated Signaling Pathways: A Unifying Hypothesis of Type 2 Diabetes. Endocr. Rev..

[25] González F., Considine R.V., Abdelhadi O.A., Acton A.J. (2018). Saturated Fat Ingestion Promotes Lipopolysaccharide-Mediated Inflammation and Insulin Resistance in Polycystic Ovary Syndrome. J. Clin. Endocrinol. Metab..

[26] Szczuko M., Zapałowska-Chwyć M., Maciejewska D., Drozd A., Starczewski A., Stachowska E. (2016). High Glycemic Index Diet in PCOS Patients. The Analysis of IGF I and TNF-α Pathways in Metabolic Disorders. Med. Hypotheses.

[27] Panieri E., Saso L. (2019). Potential Applications of NRF2 Inhibitors in Cancer Therapy. Oxidative Med. Cell. Longev..

[28] Panieri E., Buha A., Telkoparan-Akillilar P., Cevik D., Kouretas D., Veskoukis A., Skaperda Z., Tsatsakis A., Wallace D., Suzen S. (2020). Potential Applications of NRF2 Modulators in Cancer Therapy. Antioxidants.

[29] Panieri E., Telkoparan-Akillilar P., Suzen S., Saso L. (2020). The NRF2/KEAP1 Axis in the Regulation of Tumor Metabolism: Mechanisms and Therapeutic Perspectives. Biomolecules.

[30] Suzuki T., Yamamoto M. (2017). Stress-Sensing Mechanisms and the Physiological Roles of the Keap1-Nrf2 System during Cellular Stress. J. Biol. Chem..

[31] Chowdhry S., Zhang Y., McMahon M., Sutherland C., Cuadrado A., Hayes J.D. (2013). Nrf2 Is Controlled by Two Distinct β-TrCP Recognition Motifs in Its Neh6 Domain, One of Which Can Be Modulated by GSK-3 Activity. Oncogene.

[32] Wu T., Zhao F., Gao B., Tan C., Yagishita N., Nakajima T., Wong P.K., Chapman E., Fang D., Zhang D.D. (2014). Hrd1 Suppresses Nrf2-Mediated Cellular Protection during Liver Cirrhosis. Genes Dev..

[33] Rojo A.I., Rada P., Mendiola M., Ortega-Molina A., Wojdyla K., Rogowska-Wrzesinska A., Hardisson D., Serrano M., Cuadrado A. (2014). The PTEN/NRF2 Axis Promotes Human Carcinogenesis. Antioxid. Redox. Signal..

[34] Zhang C., Wang H.-J., Bao Q.-C., Wang L., Guo T.-K., Chen W.-L., Xu L.-L., Zhou H.-S., Bian J.-L., Yang Y.-R. (2016). NRF2 Promotes Breast Cancer Cell Proliferation and Metastasis by Increasing RhoA/ROCK Pathway Signal Transduction. Oncotarget.

[35] Bao L., Wu J., Dodson M., Rojo de la Vega E.M., Ning Y., Zhang Z., Yao M., Zhang D.D., Xu C., Yi X. (2017). ABCF2, an Nrf2 Target Gene, Contributes to Cisplatin Resistance in Ovarian Cancer Cells. Mol. Carcinog..

[36] Wong T.F., Yoshinaga K., Monma Y., Ito K., Niikura H., Nagase S., Yamamoto M., Yaegashi N. (2011). Association of Keap1 and Nrf2 Genetic Mutations and Polymorphisms with Endometrioid Endometrial Adenocarcinoma Survival. Int. J. Gynecol. Cancer.

[37] Shibata T., Kokubu A., Gotoh M., Ojima H., Ohta T., Yamamoto M., Hirohashi S. (2008). Genetic Alteration of Keap1 Confers Constitutive Nrf2 Activation and Resistance to Chemotherapy in Gallbladder Cancer. Gastroenterology.

[38] Nioi P., Nguyen T. (2007). A Mutation of Keap1 Found in Breast Cancer Impairs Its Ability to Repress Nrf2 Activity. Biochem. Biophys. Res. Commun.

[39] Sjöblom T., Jones S., Wood L.D., Parsons D.W., Lin J., Barber T.D., Mandelker D., Leary R.J., Ptak J., Silliman N. (2006). The Consensus Coding Sequences of Human Breast and Colorectal Cancers. Science.

[40] Chu X.-Y., Li Z.-J., Zheng Z.-W., Tao Y.-L., Zou F.-X., Yang X.-F. (2018). KEAP1/NRF2 Signaling Pathway Mutations in Cervical Cancer. Eur. Rev. Med. Pharm. Sci..

[41] Konstantinopoulos P.A., Spentzos D., Fountzilas E., Francoeur N., Sanisetty S., Grammatikos A.P., Hecht J.L., Cannistra S.A. (2011). Keap1 Mutations and Nrf2 Pathway Activation in Epithelial Ovarian Cancer. Cancer Res..

[42] Martinez V.D., Vucic E.A., Thu K.L., Hubaux R., Enfield K.S.S., Pikor L.A., Becker-Santos D.D., Brown C.J., Lam S., Lam W.L. (2015). Unique Somatic and Malignant Expression Patterns Implicate PIWI-Interacting RNAs in Cancer-Type Specific Biology. Sci Rep..

[43] Yamamoto S., Inoue J., Kawano T., Kozaki K., Omura K., Inazawa J. (2014). The Impact of MiRNA-Based Molecular Diagnostics and Treatment of NRF2-Stabilized Tumors. Mol. Cancer Res..

[44] Jiao N., Baker S.S., Nugent C.A., Tsompana M., Cai L., Wang Y., Buck M.J., Genco R.J., Baker R.D., Zhu R. (2018). Gut Microbiome May Contribute to Insulin Resistance and Systemic Inflammation in Obese Rodents: A Meta-Analysis. Physiol Genom..

[45] Zhang Z., Bai L., Guan M., Zhou X., Liang X., Lv Y., Yi H., Zhou H., Liu T., Gong P. (2021). Potential probiotics Lactobacillus casei K11 combined with plant extracts reduce markers of type 2 diabetes mellitus in mice. J. Appl. Microbiol..

[46] Bamberger C., Rossmeier A., Lechner K., Wu L., Waldmann E., Fischer S., Stark R.G., Altenhofer J., Henze K., Parhofer K.G. (2018). A Walnut-Enriched Diet Affects Gut Microbiome in Healthy Caucasian Subjects: A Randomized, Controlled Trial. Nutrients.

[47] Gomez-Arango L.F., Barrett H.L., Wilkinson S.A., Callaway L.K., McIntyre H.D., Morrison M., Dekker Nitert M. (2018). Low Dietary Fiber Intake Increases Collinsella Abundance in the Gut Microbiota of Overweight and Obese Pregnant Women. Gut Microbes.

[48] Ojo O., Feng Q.-Q., Ojo O.O., Wang X.-H. (2020). The Role of Dietary Fibre in Modulating Gut Microbiota Dysbiosis in Patients with Type 2 Diabetes: A Systematic Review and Meta-Analysis of Randomised Controlled Trials. Nutrients.

[49] den Besten G., van Eunen K., Groen A.K., Venema K., Reijngoud D.-J., Bakker B.M. (2013). The Role of Short-Chain Fatty Acids in the Interplay between Diet, Gut Microbiota, and Host Energy Metabolism. J. Lipid Res..

[50] Heimann E., Nyman M., Pålbrink A.-K., Lindkvist-Petersson K., Degerman E. (2016). Branched Short-Chain Fatty Acids Modulate Glucose and Lipid Metabolism in Primary Adipocytes. Adipocyte.

[51] Matijašić M., Meštrović T., Perić M., Čipčić Paljetak H., Panek M., Vranešić Bender D., Ljubas Kelečić D., Krznarić Ž., Verbanac D. (2016). Modulating Composition and Metabolic Activity of the Gut Microbiota in IBD Patients. Int. J. Mol. Sci.

[52] Meštrović T., Matijašić M., Perić M., Čipčić Paljetak H., Barešić A., Verbanac D. (2020). The Role of Gut, Vaginal, and Urinary Microbiome in Urinary Tract Infections: From Bench to Bedside. Diagnostics.

[53] Franks S., Kiddy D.S., Hamilton-Fairley D., Bush A., Sharp P.S., Reed M.J. (1991). The Role of Nutrition and Insulin in the Regulation of Sex Hormone Binding Globulin. J. Steroid Biochem. Mol. Biol..

[54] Tymchuk C.N., Tessler S.B., Barnard R.J. (2000). Changes in Sex Hormone-Binding Globulin, Insulin, and Serum Lipids in Postmenopausal Women on a Low-Fat, High-Fiber Diet Combined with Exercise. Nutr. Cancer.

[55] Gann P.H., Chatterton R.T., Gapstur S.M., Liu K., Garside D., Giovanazzi S., Thedford K., Van Horn L. (2003). The Effects of a Low-Fat/High-Fiber Diet on Sex Hormone Levels and Menstrual Cycling in Premenopausal Women: A 12-Month Randomized Trial (the Diet and Hormone Study). Cancer.

[56] Moran L.J., Noakes M., Clifton P.M., Tomlinson L., Galletly C., Norman R.J. (2003). Dietary Composition in Restoring Reproductive and Metabolic Physiology in Overweight Women with Polycystic Ovary Syndrome. J. Clin. Endocrinol. Metab..

[57] Szczuko M., Zapałowska-Chwyć M., Drozd A., Maciejewska D., Starczewski A., Wysokiński P., Stachowska E. (2018). Changes in the IGF-1 and TNF-α Synthesis Pathways before and after Three-Month Reduction Diet with Low Glicemic Index in Women with PCOS. Ginekol. Pol..

[58] Kazemi M., Hadi A., Pierson R.A., Lujan M.E., Zello G.A., Chilibeck P.D. (2021). Effects of Dietary Glycemic Index and Glycemic Load on Cardiometabolic and Reproductive Profiles in Women with Polycystic Ovary Syndrome: A Systematic Review and Meta-Analysis of Randomized Controlled Trials. Adv. Nutr..

[59] Wang T., Sha L., Li Y., Zhu L., Wang Z., Li K., Lu H., Bao T., Guo L., Zhang X. (2020). Dietary α-Linolenic Acid-Rich Flaxseed Oil Exerts Beneficial Effects on Polycystic Ovary Syndrome Through Sex Steroid Hormones-Microbiota-Inflammation Axis in Rats. Front. Endocrinol..

[60] Barber T.M., Kabisch S., Pfeiffer A.F.H., Weickert M.O. (2020). The Health Benefits of Dietary Fibre. Nutrients.

[61] Hoover S.E., Gower B.A., Cedillo Y.E., Chandler-Laney P.C., Deemer S.E., Goss A.M. (2021). Changes in Ghrelin and Glucagon Following a Low Glycemic Load Diet in Women with PCOS. J. Clin. Endocrinol. Metab..

[62] Akintayo C.O., Johnson A.D., Badejogbin O.C., Olaniyi K.S., Oniyide A.A., Ajadi I.O., Ojewale A.O., Adeyomoye O.I., Kayode A.B. (2021). High Fructose-Enriched Diet Synergistically Exacerbates Endocrine but Not Metabolic Changes in Letrozole-Induced Polycystic Ovarian Syndrome in Wistar Rats. Heliyon.

[63] Shang Y., Zhou H., Hu M., Feng H. (2020). Effect of Diet on Insulin Resistance in Polycystic Ovary Syndrome. J. Clin. Endocrinol. Metab..

[64] Porchia L.M., Hernandez-Garcia S.C., Gonzalez-Mejia M.E., López-Bayghen E. (2020). Diets with Lower Carbohydrate Concentrations Improve Insulin Sensitivity in Women with Polycystic Ovary Syndrome: A Meta-Analysis. Eur. J. Obs. Gynecol. Reprod. Biol..

[65] Shishehgar F., Mirmiran P., Rahmati M., Tohidi M., Ramezani Tehrani F. (2019). Does a Restricted Energy Low Glycemic Index Diet Have a Different Effect on Overweight Women with or without Polycystic Ovary Syndrome?. BMC Endocr. Disord..

[66] Paoli A., Mancin L., Giacona M.C., Bianco A., Caprio M. (2020). Effects of a Ketogenic Diet in Overweight Women with Polycystic Ovary Syndrome. J. Transl. Med..

[67] Fonseka S., Subhani B., Wijeyaratne C.N., Gawarammana I.B., Kalupahana N.S., Ratnatunga N., Rosairo S., Vithane K.P. (2019). Association between Visceral Adiposity Index, Hirsutism and Cardiometabolic Risk Factors in Women with Polycystic Ovarian Syndrome: A Cross-Sectional Study. Ceylon Med. J..

[68] Marson E.C., Delevatti R.S., Prado A.K.G., Netto N., Kruel L.F.M. (2016). Effects of Aerobic, Resistance, and Combined Exercise Training on Insulin Resistance Markers in Overweight or Obese Children and Adolescents: A Systematic Review and Meta-Analysis. Prev. Med..

[69] Patten R.K., Boyle R.A., Moholdt T., Kiel I., Hopkins W.G., Harrison C.L., Stepto N.K. (2020). Exercise Interventions in Polycystic Ovary Syndrome: A Systematic Review and Meta-Analysis. Front. Physiol..

[70] Santos I.K.D., Nunes F.A.S.d.S., Queiros V.S., Cobucci R.N., Dantas P.B., Soares G.M., Cabral B.G.d.A.T., Maranhão T.M.d.O., Dantas P.M.S. (2021). Effect of High-Intensity Interval Training on Metabolic Parameters in Women with Polycystic Ovary Syndrome: A Systematic Review and Meta-Analysis of Randomized Controlled Trials. PLoS ONE.

[71] Shele G., Genkil J., Speelman D. (2020). A Systematic Review of the Effects of Exercise on Hormones in Women with Polycystic Ovary Syndrome. J. Funct. Morphol. Kinesiol..

[72] Yang Y., Deng H., Li T., Xia M., Liu C., Bu X.-Q., Li H., Fu L.-J., Zhong Z.-H. (2021). The Mental Health of Chinese Women with Polycystic Ovary Syndrome Is Related to Sleep Disorders, Not Disease Status. J. Affect. Disord..

[73] Leproult R., Van Cauter E. (2010). Role of Sleep and Sleep Loss in Hormonal Release and Metabolism. Endocr. Dev..

[74] Donga E., Romijn J.A. (2014). Sleep Characteristics and Insulin Sensitivity in Humans. Handb. Clin. Neurol..

[75] Reutrakul S., Van Cauter E. (2018). Sleep Influences on Obesity, Insulin Resistance, and Risk of Type 2 Diabetes. Metabolism.

[76] Poroyko V.A., Carreras A., Khalyfa A., Khalyfa A.A., Leone V., Peris E., Almendros I., Gileles-Hillel A., Qiao Z., Hubert N. (2016). Chronic Sleep Disruption Alters Gut Microbiota, Induces Systemic and Adipose Tissue Inflammation and Insulin Resistance in Mice. Sci. Rep..

[77] Mojaverrostami S., Asghari N., Khamisabadi M., Heidari Khoei H. (2019). The Role of Melatonin in Polycystic Ovary Syndrome: A Review. Int. J. Reprod. Biomed..

[78] Szczuko M., Hawryłkowicz V., Kikut J., Drozd A. (2020). The Implications of Vitamin Content in the Plasma in Reference to the Parameters of Carbohydrate Metabolism and Hormone and Lipid Profiles in PCOS. J. Steroid Biochem. Mol. Biol..

[79] Szczuko M., Szydłowska I., Nawrocka-Rutkowska J. (2021). A Properly Balanced Reduction Diet and/or Supplementation Solve the Problem with the Deficiency of These Vitamins Soluble in Water in Patients with PCOS. Nutrients.

[80] Suzuki H., Kunisawa J. (2015). Vitamin-Mediated Immune Regulation in the Development of Inflammatory Diseases. Endocr. Metab. Immune Disord. Drug Targets.

[81] Wanders D., Graff E.C., White B.D., Judd R.L. (2013). Niacin Increases Adiponectin and Decreases Adipose Tissue Inflammation in High Fat Diet-Fed Mice. PLoS ONE.

[82] Esmaeilzadeh S., Gholinezhad-Chari M., Ghadimi R. (2017). The Effect of Metformin Treatment on the Serum Levels of Homocysteine, Folic Acid, and Vitamin B12 in Patients with Polycystic Ovary Syndrome. J. Hum. Reprod. Sci..

[83] DiNicolantonio J.J., Liu J., O’Keefe J.H. (2018). Thiamine and Cardiovascular Disease: A Literature Review. Prog. Cardiovasc. Dis..

[84] Eshak E.S., Arafa A.E. (2018). Thiamine Deficiency and Cardiovascular Disorders. Nutr. Metab. Cardiovasc. Dis..

[85] Taghizadeh S., Izadi A., Shirazi S., Parizad M., Pourghassem Gargari B. (2021). The Effect of Coenzyme Q10 Supplementation on Inflammatory and Endothelial Dysfunction Markers in Overweight/Obese Polycystic Ovary Syndrome Patients. Gynecol. Endocrinol..

[86] Teegarden D., Donkin S.S. (2009). Vitamin D: Emerging New Roles in Insulin Sensitivity. Nutr. Res. Rev..

[87] He C., Lin Z., Robb S.W., Ezeamama A.E. (2015). Serum Vitamin D Levels and Polycystic Ovary Syndrome: A Systematic Review and Meta-Analysis. Nutrients.

[88] Wehr E., Pieber T.R., Obermayer-Pietsch B. (2011). Effect of Vitamin D3 Treatment on Glucose Metabolism and Menstrual Frequency in Polycystic Ovary Syndrome Women: A Pilot Study. J. Endocrinol. Investig..

[89] Maktabi M., Jamilian M., Asemi Z. (2018). Magnesium-Zinc-Calcium-Vitamin D Co-Supplementation Improves Hormonal Profiles, Biomarkers of Inflammation and Oxidative Stress in Women with Polycystic Ovary Syndrome: A Randomized, Double-Blind, Placebo-Controlled Trial. Biol. Trace Elem. Res..

[90] Jamilian M., Samimi M., Mirhosseini N., Afshar Ebrahimi F., Aghadavod E., Talaee R., Jafarnejad S., Hashemi Dizaji S., Asemi Z. (2018). The Influences of Vitamin D and Omega-3 Co-Supplementation on Clinical, Metabolic and Genetic Parameters in Women with Polycystic Ovary Syndrome. J. Affect. Disord..

[91] Formuso C., Stracquadanio M., Ciotta L. (2015). Myo-Inositol vs. D-Chiro Inositol in PCOS Treatment. Minerva Ginecol..

[92] Fruzzetti F., Perini D., Russo M., Bucci F., Gadducci A. (2017). Comparison of Two Insulin Sensitizers, Metformin and Myo-Inositol, in Women with Polycystic Ovary Syndrome (PCOS). Gynecol. Endocrinol..

[93] Saleem F., Rizvi S.W. (2017). New Therapeutic Approaches in Obesity and Metabolic Syndrome Associated with Polycystic Ovary Syndrome. Cureus.

[94] Genazzani A.D., Santagni S., Ricchieri F., Campedelli A., Rattighieri E., Chierchia E., Marini G., Despini G., Prati A., Simoncini T. (2014). Myo-Inositol Modulates Insulin and Luteinizing Hormone Secretion in Normal Weight Patients with Polycystic Ovary Syndrome. J. Obs. Gynaecol. Res..

[95] Facchinetti F., Bizzarri M., Benvenga S., D’Anna R., Lanzone A., Soulage C., Di Renzo G.C., Hod M., Cavalli P., Chiu T.T. (2015). Results from the International Consensus Conference on Myo-Inositol and d-Chiro-Inositol in Obstetrics and Gynecology: The Link between Metabolic Syndrome and PCOS. Eur. J. Obs. Gynecol. Reprod. Biol..

[96] Unfer V., Nestler J.E., Kamenov Z.A., Prapas N., Facchinetti F. (2016). Effects of Inositol(s) in Women with PCOS: A Systematic Review of Randomized Controlled Trials. Int. J. Endocrinol..

[97] Li Y., Ma H., Zhang Y., Kuang H., Ng E.H.Y., Hou L., Wu X. (2013). Effect of Berberine on Insulin Resistance in Women with Polycystic Ovary Syndrome: Study Protocol for a Randomized Multicenter Controlled Trial. Trials.

[98] Rondanelli M., Infantino V., Riva A., Petrangolini G., Faliva M.A., Peroni G., Naso M., Nichetti M., Spadaccini D., Gasparri C. (2020). Polycystic Ovary Syndrome Management: A Review of the Possible Amazing Role of Berberine. Arch. Gynecol. Obs..

[99] Xiang D., Lu J., Wei C., Cai X., Wang Y., Liang Y., Xu M., Wang Z., Liu M., Wang M. (2020). Berberine Ameliorates Prenatal Dihydrotestosterone Exposure-Induced Autism-Like Behavior by Suppression of Androgen Receptor. Front. Cell Neurosci..

[100] Bertuccioli A., Moricoli S., Amatori S., Rocchi M.B.L., Vici G., Sisti D. (2020). Berberine and Dyslipidemia: Different Applications and Biopharmaceutical Formulations Without Statin-Like Molecules-A Meta-Analysis. J. Med. Food.

[101] Wei W., Zhao H., Wang A., Sui M., Liang K., Deng H., Ma Y., Zhang Y., Zhang H., Guan Y. (2012). A Clinical Study on the Short-Term Effect of Berberine in Comparison to Metformin on the Metabolic Characteristics of Women with Polycystic Ovary Syndrome. Eur. J. Endocrinol..

[102] Kuang H., Duan Y., Li D., Xu Y., Ai W., Li W., Wang Y., Liu S., Li M., Liu X. (2020). The Role of Serum Inflammatory Cytokines and Berberine in the Insulin Signaling Pathway among Women with Polycystic Ovary Syndrome. PLoS ONE.

[103] Lucidi R.S., Thyer A.C., Easton C.A., Holden A.E.C., Schenken R.S., Brzyski R.G. (2005). Effect of Chromium Supplementation on Insulin Resistance and Ovarian and Menstrual Cyclicity in Women with Polycystic Ovary Syndrome. Fertil. Steril..

[104] Tang X.-L., Sun Z., Gong L. (2018). Chromium Supplementation in Women with Polycystic Ovary Syndrome: Systematic Review and Meta-Analysis. J. Obs. Gynaecol. Res..

[105] Fazelian S., Rouhani M.H., Bank S.S., Amani R. (2017). Chromium Supplementation and Polycystic Ovary Syndrome: A Systematic Review and Meta-Analysis. J. Trace Elem. Med. Biol..

[106] Ashoush S., Abou-Gamrah A., Bayoumy H., Othman N. (2016). Chromium Picolinate Reduces Insulin Resistance in Polycystic Ovary Syndrome: Randomized Controlled Trial. J. Obs. Gynaecol. Res..

[107] Piotrowska A., Pilch W., Czerwińska-Ledwig O., Zuziak R., Siwek A., Wolak M., Nowak G. (2019). The Possibilities of Using Chromium Salts as an Agent Supporting Treatment of Polycystic Ovary Syndrome. Biol. Trace Elem. Res..

[108] Maxel T., Svendsen P.F., Smidt K., Lauridsen J.K., Brock B., Pedersen S.B., Rungby J., Larsen A. (2017). Expression Patterns and Correlations with Metabolic Markers of Zinc Transporters ZIP14 and ZNT1 in Obesity and Polycystic Ovary Syndrome. Front. Endocrinol..

[109] Nasiadek M., Stragierowicz J., Klimczak M., Kilanowicz A. (2020). The Role of Zinc in Selected Female Reproductive System Disorders. Nutrients.

[110] Guler I., Himmetoglu O., Turp A., Erdem A., Erdem M., Onan M.A., Taskiran C., Taslipinar M.Y., Guner H. (2014). Zinc and Homocysteine Levels in Polycystic Ovarian Syndrome Patients with Insulin Resistance. Biol. Trace Elem. Res..

[111] Coskun A., Arikan T., Kilinc M., Arikan D.C., Ekerbiçer H.Ç. (2013). Plasma Selenium Levels in Turkish Women with Polycystic Ovary Syndrome. Eur. J. Obs. Gynecol. Reprod. Biol..

[112] Michael P.J., Stepanić V., Nadja T., Panek M., Verbanac D. (2019). Mild Plant and Dietary Immunomodulators. Nijkamp Parnham’s Princ. Immunopharmacol..

[113] Ma X., Weng X., Hu X., Wang Q., Tian Y., Ding Y., Zhang C. (2019). Roles of Different N-3/n-6 PUFA Ratios in Ovarian Cell Development and Steroidogenesis in PCOS Rats. Food. Funct..

[114] Popova A., Mihaylova D. (2018). A Review of the Medicinal Plants in Bulgaria: Collection, Storage, And Extraction Techniques. Asian J. Pharm. Clin. Res..

[115] Ashkar F., Rezaei S., Salahshoornezhad S., Vahid F., Gholamalizadeh M., Dahka S.M., Doaei S. (2020). The Role of Medicinal Herbs in Treatment of Insulin Resistance in Patients with Polycystic Ovary Syndrome: A Literature Review. Biomol. Concepts.

[116] Tehrani H.G., Allahdadian M., Zarre F., Ranjbar H., Allahdadian F. (2017). Effect of Green Tea on Metabolic and Hormonal Aspect of Polycystic Ovarian Syndrome in Overweight and Obese Women Suffering from Polycystic Ovarian Syndrome: A Clinical Trial. J. Educ. Health Promot..

[117] Haj-Husein I., Tukan S., Alkazaleh F. (2016). The Effect of Marjoram (Origanum Majorana) Tea on the Hormonal Profile of Women with Polycystic Ovary Syndrome: A Randomised Controlled Pilot Study. J. Hum. Nutr. Diet..

[118] Rababa’h A.M., Matani B.R., Ababneh M.A. (2020). The Ameliorative Effects of Marjoram in Dehydroepiandrosterone Induced Polycystic Ovary Syndrome in Rats. Life Sci..

[119] Grant P. (2010). Spearmint Herbal Tea Has Significant Anti-Androgen Effects in Polycystic Ovarian Syndrome. A Randomized Controlled Trial. Phytother. Res..

[120] Sadeghi Ataabadi M., Alaee S., Bagheri M.J., Bahmanpoor S. (2017). Role of Essential Oil of Mentha Spicata (Spearmint) in Addressing Reverse Hormonal and Folliculogenesis Disturbances in a Polycystic Ovarian Syndrome in a Rat Model. Adv. Pharm. Bull..

[121] Sabbadin C., Bordin L., Donà G., Manso J., Avruscio G., Armanini D. (2019). Licorice: From Pseudohyperaldosteronism to Therapeutic Uses. Front. Endocrinol..

[122] Arentz S., Abbott J.A., Smith C.A., Bensoussan A. (2014). Herbal Medicine for the Management of Polycystic Ovary Syndrome (PCOS) and Associated Oligo/Amenorrhoea and Hyperandrogenism; a Review of the Laboratory Evidence for Effects with Corroborative Clinical Findings. BMC Complement. Altern Med..

[123] Adamczak M., Wiecek A. (2020). Food Products That May Cause an Increase in Blood Pressure. Curr. Hypertens. Rep..

[124] Dhariwala M.Y., Ravikumar P. (2019). An Overview of Herbal Alternatives in Androgenetic Alopecia. J. Cosmet Derm..

[125] Kakadia N., Patel P., Deshpande S., Shah G. (2019). Effect of Vitex Negundo L. Seeds in Letrozole Induced Polycystic Ovarian Syndrome. J. Tradit Complement. Med..

[126] Mehraban M., Jelodar G., Rahmanifar F. (2020). A Combination of Spearmint and Flaxseed Extract Improved Endocrine and Histomorphology of Ovary in Experimental PCOS. J. Ovarian Res..

[127] Brooks J.D., Thompson L.U. (2005). Mammalian Lignans and Genistein Decrease the Activities of Aromatase and 17beta-Hydroxysteroid Dehydrogenase in MCF-7 Cells. J. Steroid Biochem. Mol. Biol..

[128] Heshmati J., Moini A., Sepidarkish M., Morvaridzadeh M., Salehi M., Palmowski A., Mojtahedi M.F., Shidfar F. (2021). Effects of Curcumin Supplementation on Blood Glucose, Insulin Resistance and Androgens in Patients with Polycystic Ovary Syndrome: A Randomized Double-Blind Placebo-Controlled Clinical Trial. Phytomedicine.

[129] Marmitt D.J., Shahrajabian M.H., Goettert M.I., Rempel C. (2021). Clinical Trials with Plants in Diabetes Mellitus Therapy: A Systematic Review. Expert Rev. Clin. Pharm..

[130] Heshmati J., Golab F., Morvaridzadeh M., Potter E., Akbari-Fakhrabadi M., Farsi F., Tanbakooei S., Shidfar F. (2020). The Effects of Curcumin Supplementation on Oxidative Stress, Sirtuin-1 and Peroxisome Proliferator Activated Receptor γ Coactivator 1α Gene Expression in Polycystic Ovarian Syndrome (PCOS) Patients: A Randomized Placebo-Controlled Clinical Trial. Diabetes Metab. Syndr..

[131] Yuandani I.J., Rohani A.S., Sumantri I.B. (2021). Immunomodulatory Effects and Mechanisms of Curcuma Species and Their Bioactive Compounds: A Review. Front. Pharm..

[132] Chowdhury I., Banerjee S., Driss A., Xu W., Mehrabi S., Nezhat C., Sidell N., Taylor R.N., Thompson W.E. (2019). Curcumin Attenuates Proangiogenic and Proinflammatory Factors in Human Eutopic Endometrial Stromal Cells through the NF-ΚB Signaling Pathway. J. Cell Physiol.

[133] İşler S.C., Demircan S., Çakarer S., Çebi Z., Keskin C., Soluk M., Yüzbaşıoğlu E. (2010). Effects of Folk Medicinal Plant Extract Ankaferd Blood Stopper® on Early Bone Healing. J. Appl. Oral Sci..

[134] Ziaei R., Foshati S., Hadi A., Kermani M.A.H., Ghavami A., Clark C.C.T., Tarrahi M.J. (2020). The Effect of Nettle (Urtica Dioica) Supplementation on the Glycemic Control of Patients with Type 2 Diabetes Mellitus: A Systematic Review and Meta-Analysis. Phytother. Res..

[135] Sarma Kataki M., Murugamani V., Rajkumari A., Singh Mehra P., Awasthi D., Shankar Yadav R. (2012). Antioxidant, Hepatoprotective, and Anthelmintic Activities of Methanol Extract of Urtica Dioica L. Leaves. Pharm. Crop..

[136] Ferro D., Baratta F., Pastori D., Cocomello N., Colantoni A., Angelico F., Del Ben M. (2020). New Insights into the Pathogenesis of Non-Alcoholic Fatty Liver Disease: Gut-Derived Lipopolysaccharides and Oxidative Stress. Nutrients.

[137] Wat E., Wang Y., Chan K., Law H.W., Koon C.M., Lau K.M., Leung P.C., Yan C., Lau C.B.S. (2018). An in Vitro and in Vivo Study of a 4-Herb Formula on the Management of Diet-Induced Metabolic Syndrome. Phytomedicine.

[138] MacDonald-Ramos K., Michán L., Martínez-Ibarra A., Cerbón M. (2020). Silymarin Is an Ally against Insulin Resistance: A Review. Ann. Hepatol..

[139] Oppedisano F., Muscoli C., Musolino V., Carresi C., Macrì R., Giancotta C., Bosco F., Maiuolo J., Scarano F., Paone S. (2020). The Protective Effect of Cynara Cardunculus Extract in Diet-Induced NAFLD: Involvement of OCTN1 and OCTN2 Transporter Subfamily. Nutrients.

[140] Zhao Y.-M., Wang C., Zhang R., Hou X.-J., Zhao F., Zhang J.-J., Wang C. (2020). [Study on literature of artichoke and properties of traditional Chinese medicine]. Zhongguo Zhong Yao Za Zhi.

[141] Park S., Kim D.S., Wu X., J Yi Q. (2018). Mulberry and Dandelion Water Extracts Prevent Alcohol-Induced Steatosis with Alleviating Gut Microbiome Dysbiosis. Exp. Biol. Med..

[142] Azizi N., Amini M.R., Djafarian K., Shab-Bidar S. (2021). The Effects of Nigella Sativa Supplementation on Liver Enzymes Levels: A Systematic Review and Meta-Analysis of Randomized Controlled Trials. Clin. Nutr. Res..

